# Superficial myofibroblastoma of the lower female genital tract with description of the MRI features

**DOI:** 10.1259/bjrcr.20160052

**Published:** 2016-09-02

**Authors:** Sarah Ann Smith, Victoria Doyle, Elizabeth Rutherford, Victoria Elliot, Richard Murray Blaquiere

**Affiliations:** ^1^Department of Radiology, University Hospital Southampton NHS Foundation Trust, Southampton, UK; ^2^Department of Pathology, University Hospital Southampton NHS Foundation Trust, Southampton, UK

## Abstract

Superficial myofibroblastomas of the lower female genital tract are an unusual type of benign mesenchymal tumour. To the authors’ knowledge, there has been no previous imaging description of a superficial myofibroblastoma in the literature. Here, we describe a case that presented with symptoms consistent with vaginal prolapse. However, a mass was palpable on clinical examination with unusual features on MRI. Following surgery, the histopathological features were considered consistent with superficial myofibroblastoma. By presenting the MRI and histological findings, we aim to raise awareness about this lesion so that it may be considered in the differential diagnosis of a vaginal mass.

## Background

MRI of vaginal lesions can pose a challenge to radiologists owing to the broad range of tissues in an anatomically small region. Various features should be considered when forming a differential diagnosis, including precise anatomical location, signal characteristics, enhancement and involvement of other anatomical structures. Clinical examination findings and patient history are critically important but despite this information, it may still be difficult to reach a specific diagnosis, and multidisciplinary team (MDT) discussion is required to guide appropriate management thereafter.

## Case

A 73-year-old female was referred to our Trust by her general practitioner with a 5-month history of a painless vaginal mass, which extruded from the introitus on straining, but was otherwise asymptomatic. This was originally thought to be a vaginal prolapse; however, examination revealed a soft, well-defined pink mass occupying the upper vagina and an MRI of the pelvis was requested for further characterization.

**Figure 1.**
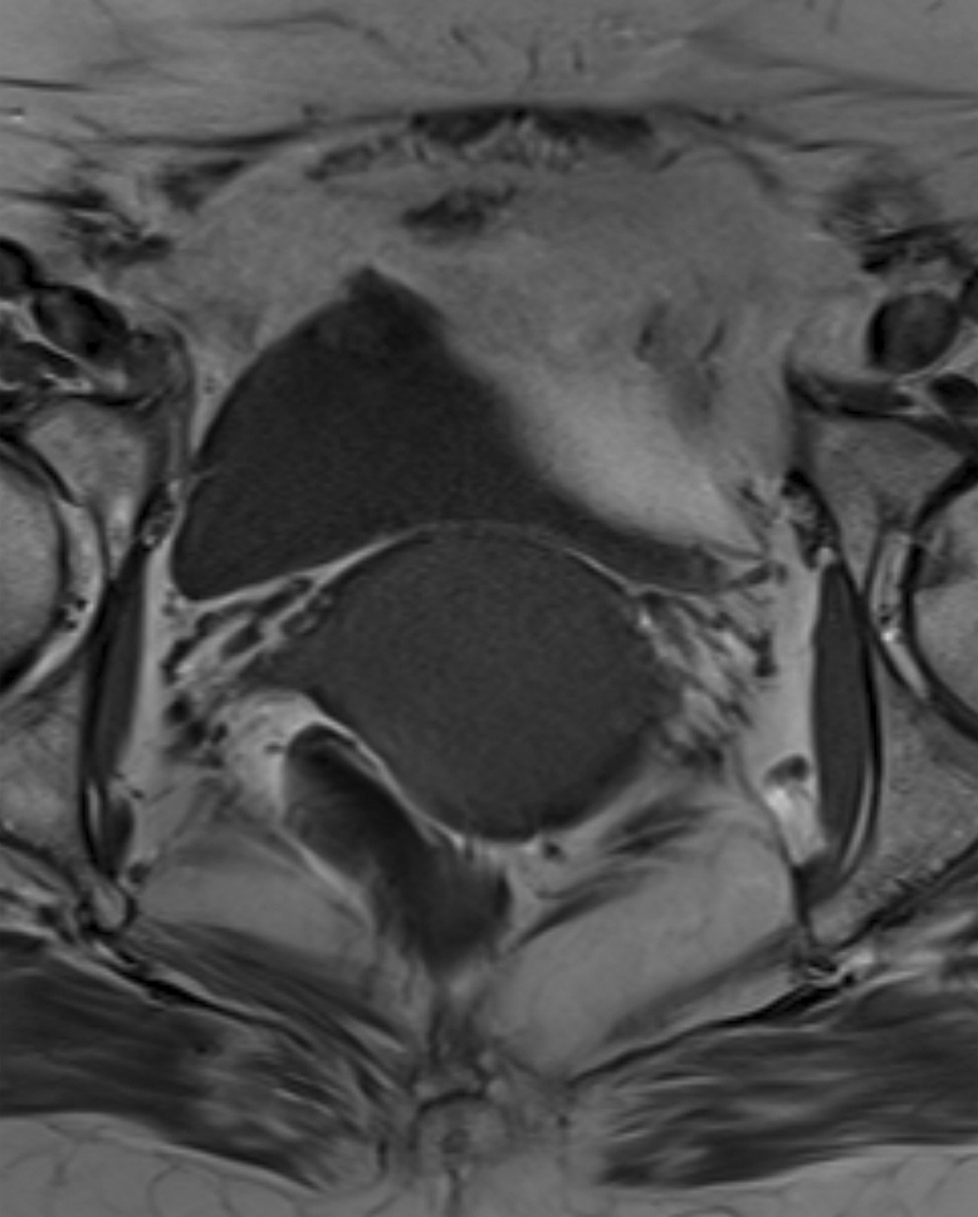
*T*_1_ weighted axial section of the pelvis. The well-circumscribed rounded mass in the region of the upper vagina demonstrates intermediate *T*_1_ signal.

**Figure 2.**
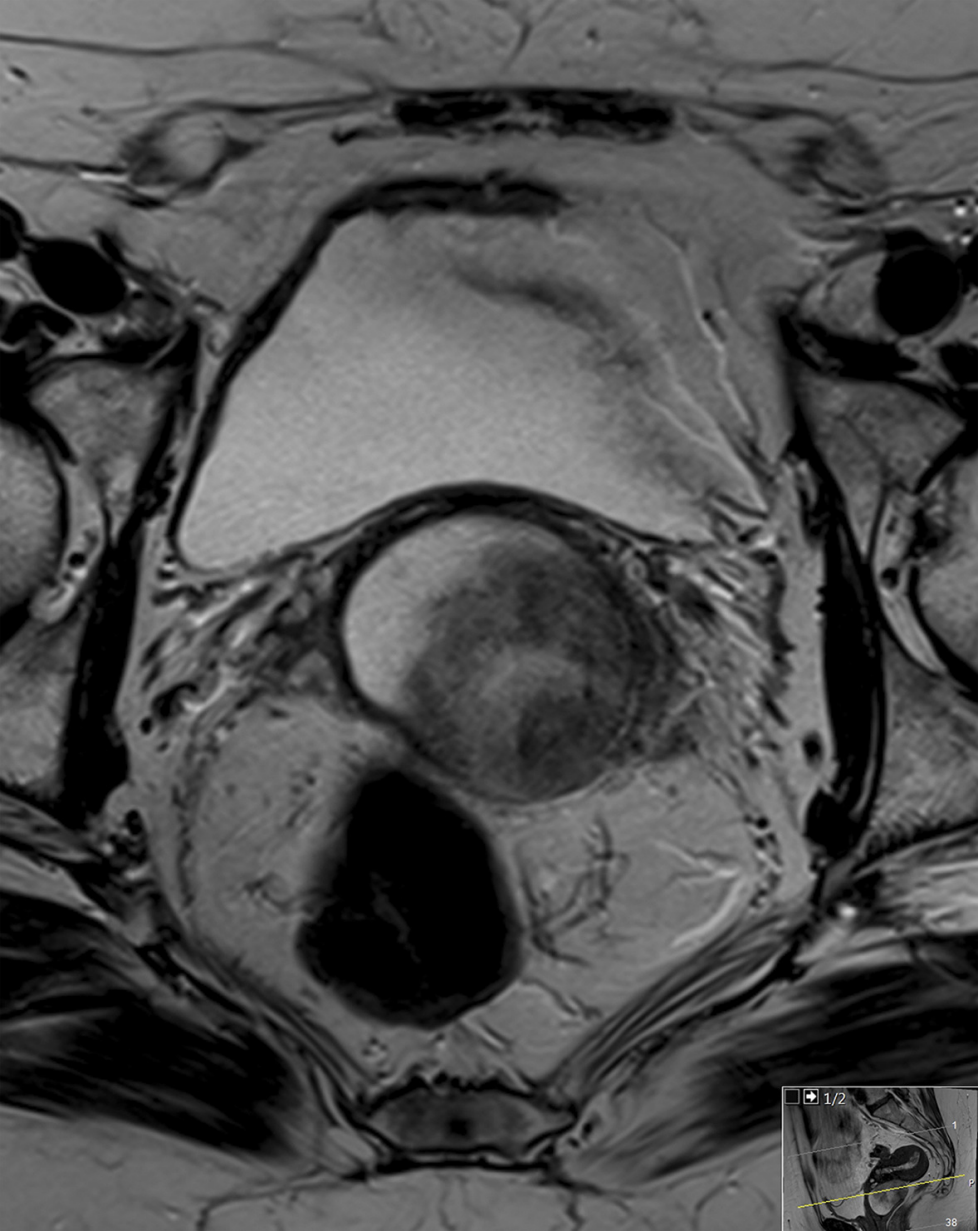
*T*_2_ weighted axial section of the pelvis with a small field of view. The well-circumscribed rounded mass in the region of the upper vagina demonstrates heterogeneous *T*_2 _signal. Loss of left posterolateral outer vaginal margin and signal change in the left paravaginal fat was noted.

**Figure 3.**
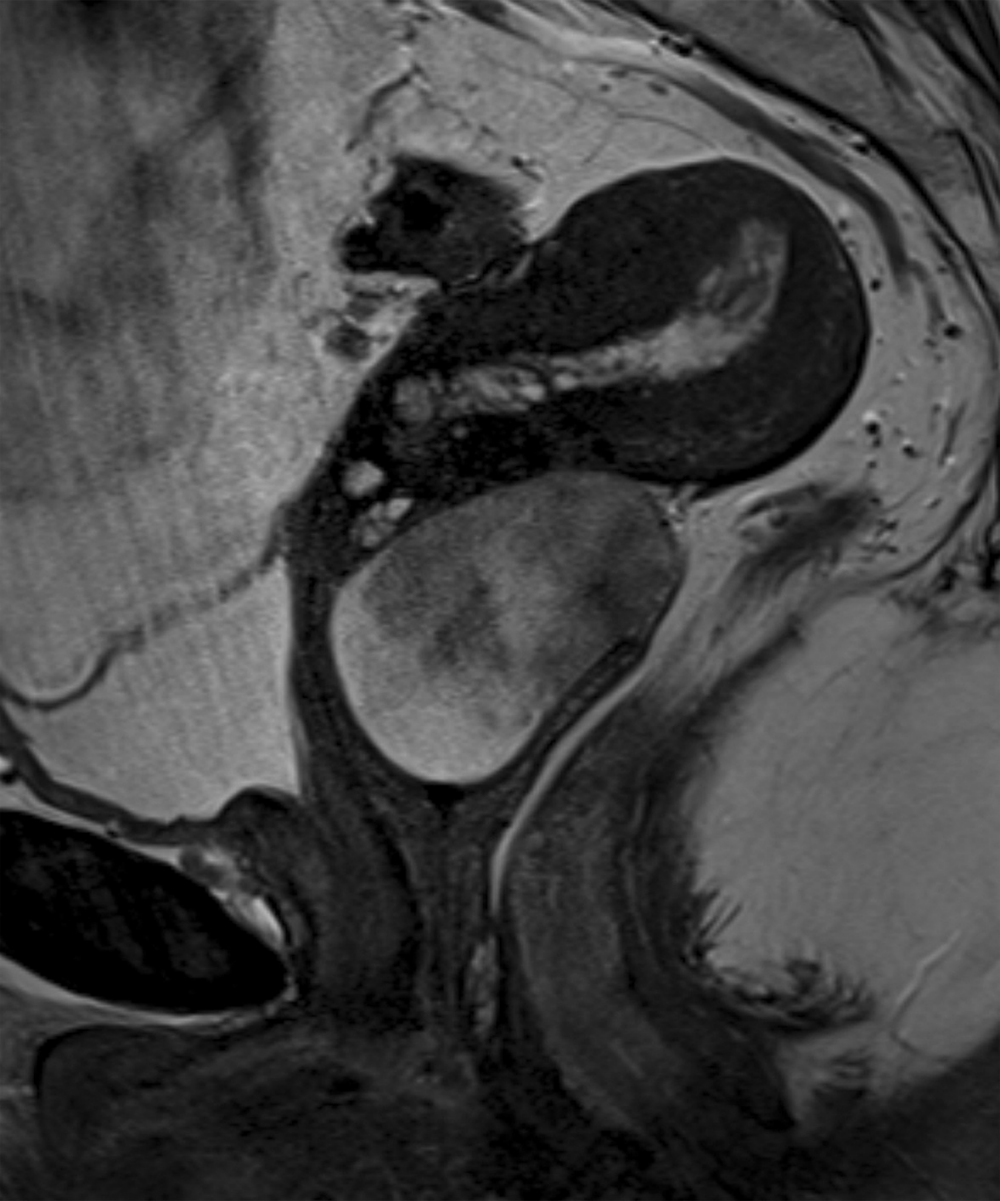
*T*_2_ weighted sagittal section of the pelvis. Discrete zones of low and high signal are demonstrated within the lesion. The mass appears to be arising within the posterior vaginal wall with displacement of the vaginal lumen anteriorly.

**Figure 4.**
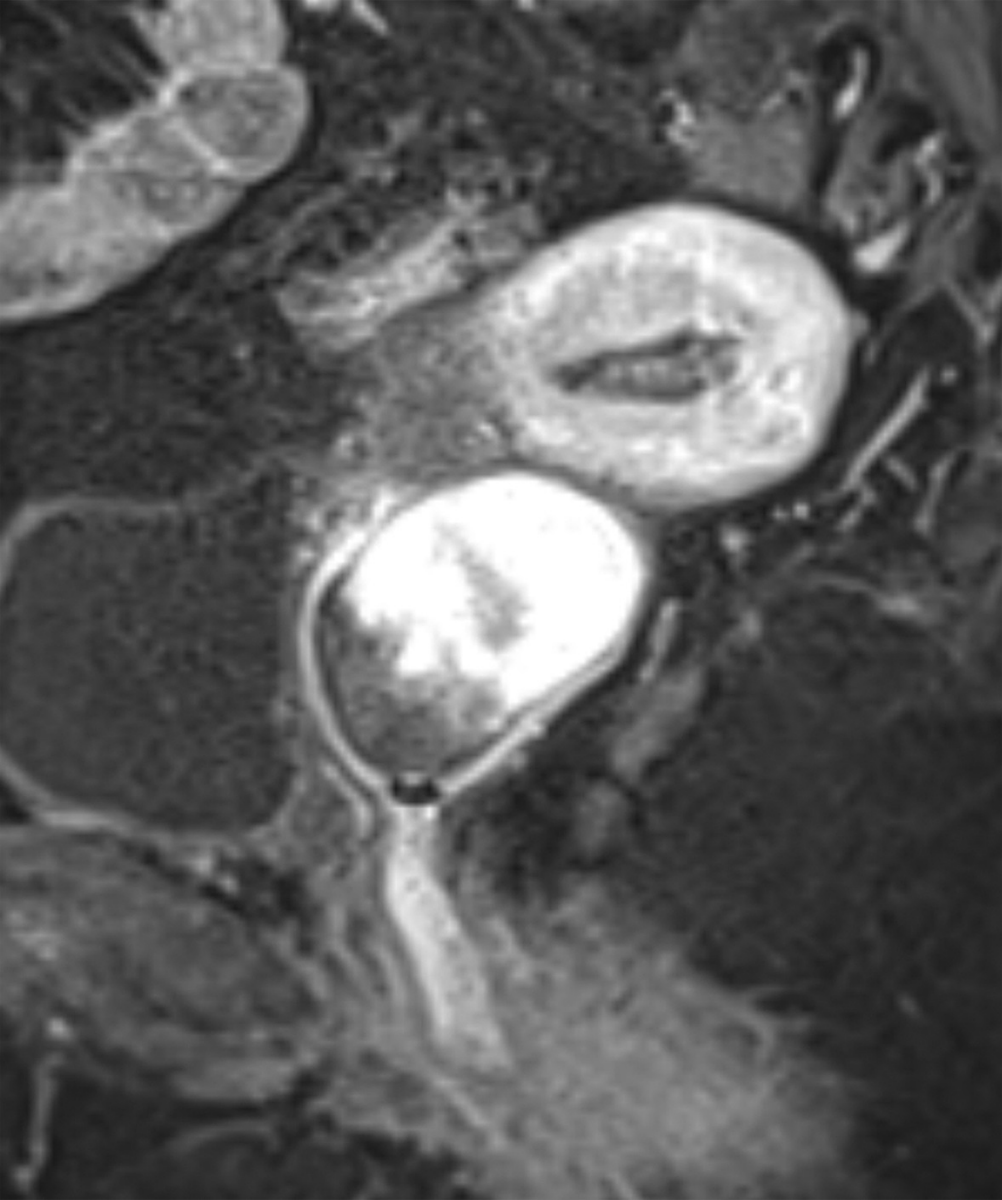
Sagittal *T*_1 _ weighted image with fat saturation and gadolinium contrast enhancement. The area of low *T*_2_ signal noted within the lesion in figure 3 demonstrates avid contrast enhancement and corresponds histologically to a hypercellular area with high collagen content. The high *T*_2_ signal area does not enhance and corresponds to paucicellular myxoid tissue.

MRI was performed using a 3.0 T system utilizing axial *T*_1_ weighted fast spin echo; small field of view axial, coronal and sagittal *T*_2_ weighted fast spin echo; and *T*_1_ weighted fat-saturated sagittal images before and after gadolinium contrast administration, obtained in the arterial and portal venous phases. Diffusion-weighted imaging was also acquired. The images demonstrated a 47 × 40 × 44 mm well-circumscribed, oval mass in the upper vagina. On the *T*_1_ weighted images, the signal intensity of the abnormality was intermediate, similar to that of the skeletal muscle (Figure 1). However, on *T*_2_ imaging, there were discrete zones within the lesion; the anteroinferior aspect was of high *T*_2_ signal with no enhancement, whereas the posterosuperior aspect was of low *T*_2_ signal with avid enhancement (Figures 2–4). There was no restricted diffusion. The posterior wall of the retroverted uterus was demonstrated to abut the superior surface of the lesion and the vaginal lumen was deviated anteriorly. Normal vaginal wall was seen to extend around the lesion’s anterior and posteroinferior surfaces. The lesion appeared to be arising within the left posterolateral vaginal wall and there were areas of loss of definition of the outer margin of the vagina. There was no involvement of the rectum, urethra or bladder; however, there were hazy low *T*_1_ and *T*_2_ signal changes in the left paravaginal fat.

**Figure 5.**
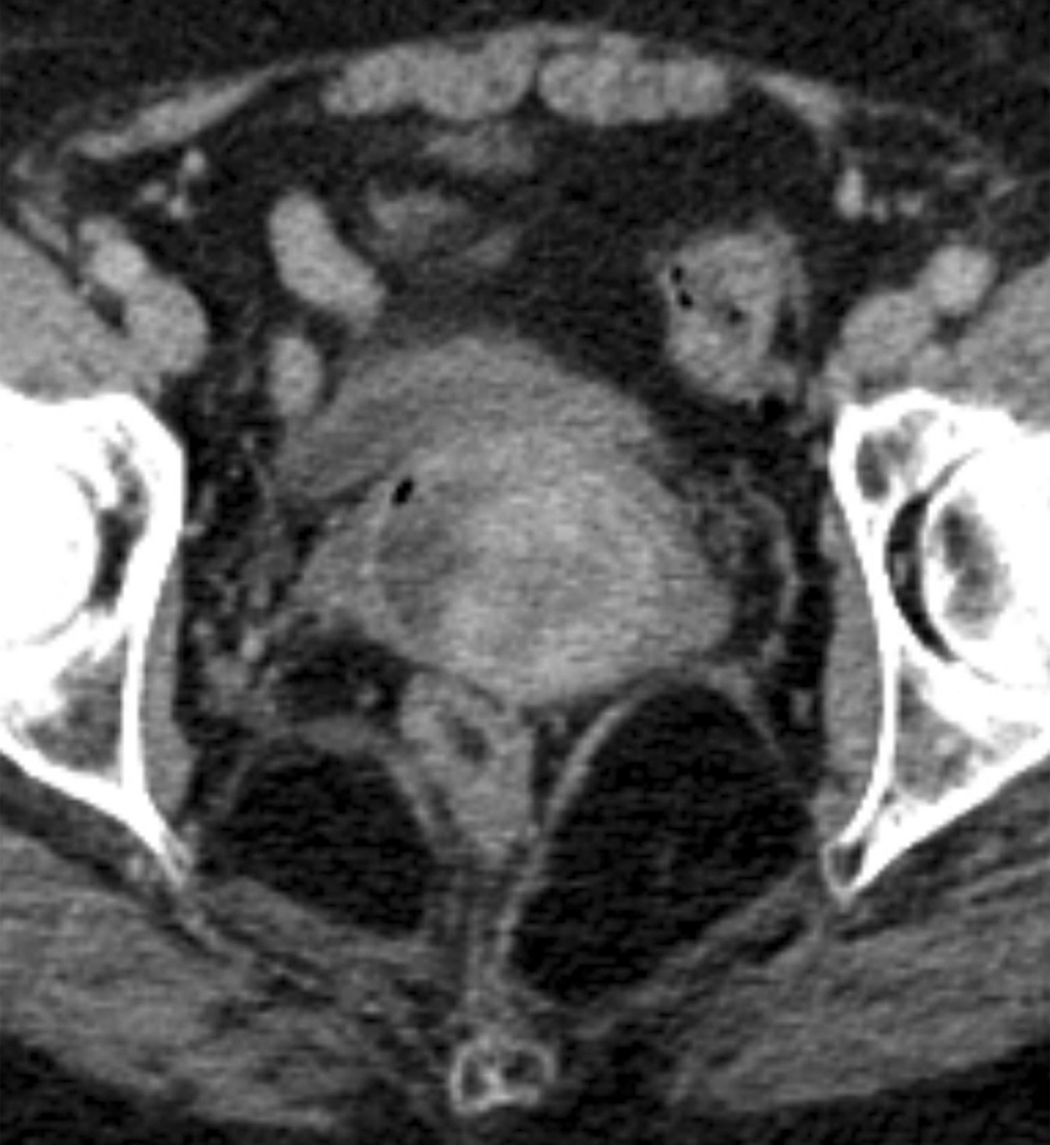
Axial portal venous phase contrast-enhanced CT image of the upper vagina demonstrating a mixed attenuation mass with patchy avid contrast enhancement.

Through a MDT discussion, it was agreed that owing to the suspicious imaging features of enhancement and tissue inhomogeneity, a staging portal venous phase CT scan was required to look for evidence of metastatic spread. Again, the vaginal lesion demonstrated fluid and soft tissue attenuation areas with regions of enhancement (Figure 5). Significantly, there was no evidence of distant spread or lymph node enlargement. After further MDT discussion, the mass was still thought to be suspicious for malignancy and the patient underwent surgery.

The uterus, ovaries, cervix and upper vagina were removed en bloc and macroscopic examination revealed a well-circumscribed 45 mm polypoid mass arising from the paracervical upper vaginal tissue. The cut surface of the lesion was fleshy grey and white in colour, and was mainly solid in nature.

**Figure 6.**
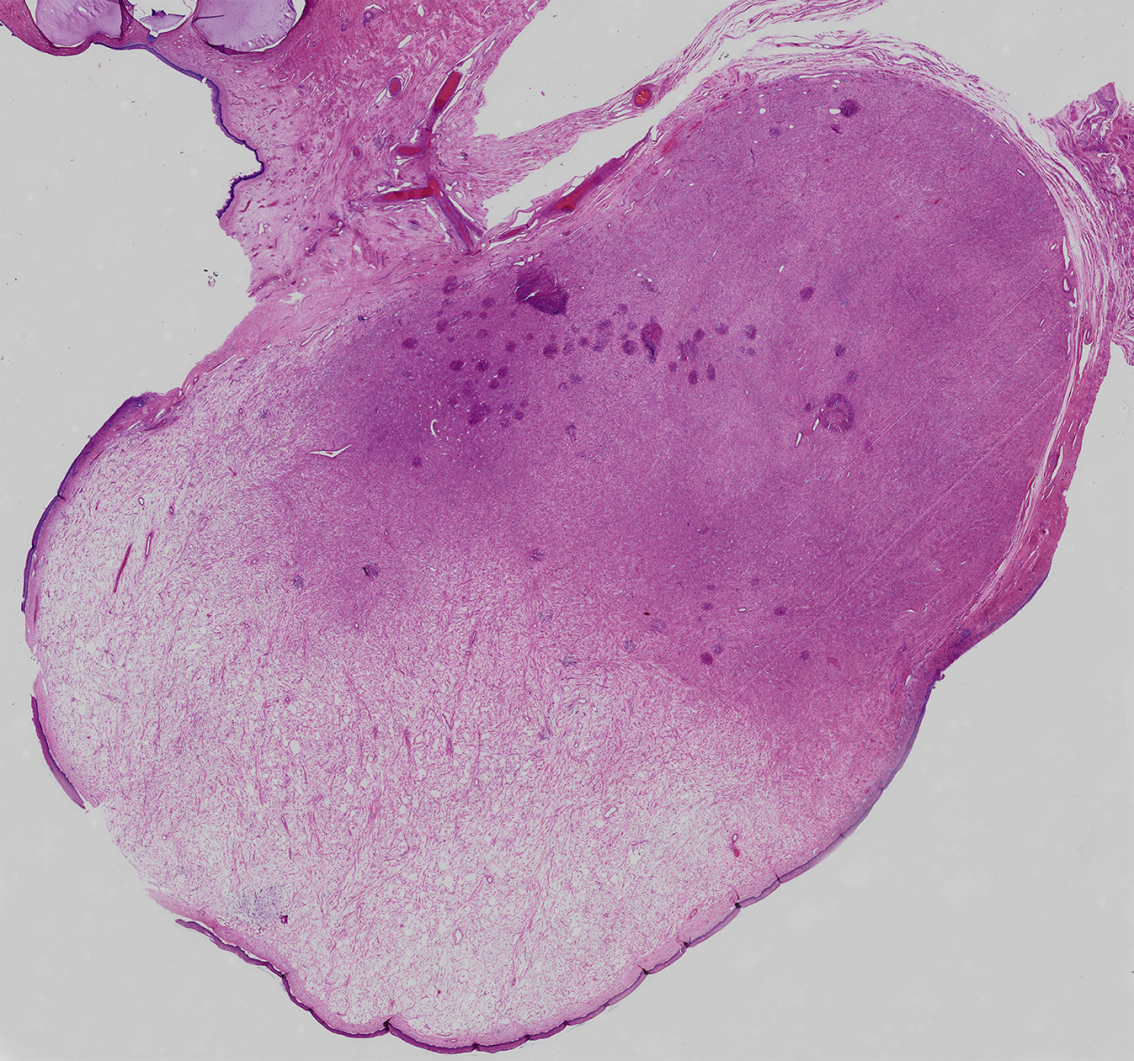
Haematoxylin and eosin preparation of the vaginal mass on a large block mount with ×2 magnification. The deep pink hypercellular area contains spindle cells set in dense collagen, whereas the paler area reflects an area of myxoid change with few cells. This correlates precisely with the appearances in Figures 3 and 4.

Microscopic examination (Figure 6) revealed an unencapsulated lesion with a spindle cell morphology arranged occasionally in fascicles. Beneath the surface epithelium, there was a grenz zone. The spindle cells were set within finely collagenized stroma and were bland in nature, with no conspicuous mitoses identified. Areas of oedema and myxoid change were also present, with no evidence of haemorrhage or necrosis.

Immunohistochemistry demonstrated that the lesional cells expressed desmin, vimentin, oestrogen and progesterone receptors. The Ki67 proliferation index was low. Immunohistochemistry for MNF116, alpha smooth muscle antigen, smooth muscle myosin, h-caldesmon, S100 and CD34 were negative in the lesional cells.

The morphological and immunohistochemical profile was considered consistent with a superficial cervicovaginal myofibroblastoma, which is also known as superficial myofibroblastoma of the lower female genital tract.

## Discussion

Superficial myofibroblastoma of the lower female genital tract is a rare tumour, typically exhibiting benign behaviour. These lesions are part of a larger group of mesenchymal vulvovaginal neoplasms, which also include angiomyofibroblastoma, cellular angiofibroma and angiomyxoma. There is considerable overlap of histological features within this group of morphologically bland proliferations.^1,2^ The origin of this group of lesions is not fully established but they are thought most likely to arise from specialized stromal cells of the lower female genital tract. In the case of superficial myofibroblastoma, an association with tamoxifen use has been described.^3^ Interestingly, the case described completed a 5-year course of tamoxifen, which finished 2 years before presentation. No relation to a viral cause has been identified.^4^

A review of 14 reported cases of superficial myofibroblastoma by Laskin et al^1^ found that the age range of affected females was 40–74 years, with a median age of 58 years. The review also found that tumours were well circumscribed and their size ranged from 1.0–6.5 cm, with a mean of 3 cm. Macroscopically, they were polypoidal or nodular in shape and were soft to rubbery, with a glistening mucoid or fleshy cut surface. Microscopically, they contained spindle and stellate cells in a collagenous stroma with myxoid and oedematous foci. They stained positive for oestrogen, progesterone, desmin, CD34 and vimentin. Follow-up of 11 of the 14 cases over 1–20 years found no instance of metastatic spread or local recurrence following excision. There has only been one reported case in the literature of local recurrence, which occurred 9 years following local excision.^5^

The radiological challenge of this case was a mixed signal vaginal lesion with features that are not commonly encountered. The well-circumscribed mass that appeared to be arising from the left posterolateral wall of the vagina had areas showing solid and fluid signal, with evidence of enhancement of the solid areas.

Other well-defined benign lesions that share a similar anatomical location include Nabothian cysts, a common lesion arising from the cervix, and Gartner duct cysts, which most frequently arise from the anterolateral wall of the upper vagina. If the lesion is found at a lower level in the vagina, other female urogenital tract lesions that could be considered include Bartholin cysts, Skene duct cysts, urethral diverticula and urethroceles. An inclusion cyst would be considered if there was a history of trauma, childbirth or surgery. These frequently encountered fluid signal lesions may have heterogeneous signal characteristics if there is secondary infection or haemorrhage; however, they would not demonstrate internal enhancement.

Degenerating leiomyomas that arise from the cervix, those that have prolapsed through the cervical os or, less commonly, those arising within the vagina may demonstrate the imaging features described here; however, pain would be a frequent clinical feature. Endometriomas may rarely arise in the vagina and their contents would have signal characteristics consistent with blood degradation products and would be more commonly seen in premenopausal females. There have been only a few reported cases of extragastrointestinal stromal tumours arising from the rectovaginal septum.^6^ As at other sites, appearances can be variable with haemorrhagic, necrotic and cystic elements. Solid components typically demonstrate high *T*_2_ and low *T*_1_ signal with contrast enhancement, and more aggressive lesions can infiltrate adjacent structures.

Malignant pathology should also be considered in the differential diagnosis of a vaginal mass. Metastases to the vagina are much more common than primary vaginal tumours, accounting for 80% of all vaginal malignancies. Vaginal involvement is frequently occurs owing to contiguous spread from a pelvic primary (ovarian, cervical, endometrial or rectal), which is usually apparent on imaging with signal characteristics corresponding to the primary tumour. Primary vaginal carcinoma is rare, and includes several cell types, of which squamous cell carcinoma is the most common (85%), followed by adenocarcinoma (9%), melanoma (3%) and sarcoma (<3%). These lesions are usually ill-defined and infiltrative, unlike the described well-circumscribed lesion, where the vaginal wall was largely intact. Squamous cell carcinoma most commonly arises from the posterior wall of the upper one-third of the vagina and shows intermediate signal intensity on *T*_2_ and hypointense signal on *T*_1_ sequences. Adenocarcinoma shares similar signal characteristics to squamous cell carcinoma but more commonly arises in the anterior wall of the upper one-third of the vagina and can appear lobulated or circumferential. Melanoma, as at other sites, demonstrates hyperintense signals on *T*_1_ and hypointense signals on *T*_2_ sequences, and tends to arise in the lower one-third of the vagina.^7^ Case reports on vaginal leiomyosarcomas are limited, but these lesions are thought to most commonly arise from the submucosa of the rectovaginal septum, protruding into the upper one-third of the vagina. The literature suggests that imaging findings are similar to those found elsewhere, with heterogeneous *T*_1_ and *T*_2_ signals owing to necrotic, cystic or haemorrhagic components. Enhancement has been described as early and heterogeneous, and the lesions are locally infiltrative.^7,8^ Spindle cell synovial sarcoma is a rare but aggressive sarcoma subtype that most commonly arises within the extremities, and rarely within the vagina. There are limited radiological descriptions of vaginal spindle cell synovial sarcoma in the literature but it is widely believed that features do mirror those from other sites, given that identical pathology has been reported.^7,9^ Spindle cell synovial sarcomas of the extremities are well defined and tend to displace rather than invade adjacent structures. Small lesions are mainly cystic; however, larger lesions can demonstrate necrosis and thus be heterogeneous on *T*_1_ and *T*_2_ sequences. Septation and loculation have been reported and up to 30% can demonstrate calcification. Contrast enhancement tends to be early and heterogeneous.^7,9^

Another important entity occurring in the lower female genital tract is aggressive angiomyxoma, which is a mesenchymal tumour that occurs in premenopausal females, with the propensity to recur and rarely metastasize. The MRI signal characteristics would be iso- to hypointense on *T*_1_ and predominantly hyperintense on *T*_2_ sequences, with swirling and enhancing central soft tissue.^10^

The histology of the lesion described in this case shows that the tumour is composed of two discrete components (Figure 6). The paler zone on the histological image corresponds to paucicellular areas with myxoid change in the stroma and shows high *T*_2_ signal areas with poor enhancement (Figures 3 and 4). The deep pink areas are hypercellular with numerous spindle cells set in dense collagen, reflected as low *T*_2_ signal avidly enhancing area. Geng et al^11^ made a similar observation when discussing the imaging features in a report of an angiomyofibroblastoma, another type of mesenchymal tumour. The similarity in MRI signal characteristics reflects similar variably dense areas within these proliferations. Angiomyofibroblastomas most commonly occur in the vulval region of reproductive and early postmenopausal females, but can occur anywhere in the female urogenital tract, as well as the scrotum and inguinal region in males, and typically also behave in a benign manner.^12^

One of the radiological features of this case was the patchy loss of definition of the outer vaginal margin and so direct infiltration could not be excluded; however, the histology did not suggest extravaginal invasion. The haziness within the paravaginal fat was of different imaging characteristic to that within the lesion, and so was thought likely to be due to tissue changes from recurring prolapse of the mass. Considering the differential diagnoses and the imaging features described, en bloc resection is advocated to ensure complete excision.

## Conclusions

We have described a case of superficial myofibroblastoma of the lower female genital tract, a relatively rare tumour with benign behaviour, for which there has been no previous discussion of imaging features. The lesion demonstrates intermediate *T*_1_ signal and heterogeneous *T*_2_ signal on MRI, with avid enhancement of the low *T*_2_ signal regions reflecting a hypercellular region and poor enhancement of high *T*_2_ signal regions reflecting paucicellular tissue. The differential diagnosis for a vaginal lesion is broad, and these specific radiological features overlap with those of more aggressive aetiology, so en bloc resection is advocated. With complete excision, there is a low chance of local recurrence and no known malignant potential. Mesenchymal proliferations should be considered in the differential diagnosis of a mass of the lower female genital tract.

## Learning points

1. Enhancing components within a mixed signal mass may not always signify malignancy.
2. Superficial myofibroblastomas of the lower female genital tract arise from the superficial tissues of the cervix, vagina and vulva, and can present with symptoms of prolapse. They are typically benign and do not usually recur after excision.
3. Superficial myofibroblastomas are well defined and demonstrate intermediate *T*_1_ and heterogeneous *T*_2_ signals. The low *T*_2_ signal areas enhance whereas high *T*_2_ signal areas do not enhance, correlating with histopathological findings within these mesenchymal tumours.

## Consent

Written informed consent for the case to be published (including images, case history and data) was obtained from the patient(s) for publication of this case report.
